# Low back pain patterns over one year among 842 workers in the DPhacto study and predictors for chronicity based on repetitive measurements

**DOI:** 10.1186/s12891-016-1307-1

**Published:** 2016-11-03

**Authors:** Julie Lagersted-Olsen, Hans Bay, Marie Birk Jørgensen, Andreas Holtermann, Karen Søgaard

**Affiliations:** 1 The National Research Centre for the Working Environment (NRCWE), Lersø Parkalle 105, 2100 Copenhagen Ø, Denmark; 2 University of Southern Denmark, Campusvej 55, 5230 Odense M, Denmark

**Keywords:** Factor analysis, Low back pain, Repeated measures, Risk factors, Text messages, Visual pain mapping

## Abstract

**Background:**

Low back pain (LBP) occurrence and intensity are considered to fluctuate over time, requiring frequent repetitive assessments to capture its true time pattern. Text messages makes frequent reporting of LBP feasible, which enables investigation of 1) the time pattern of LBP, and 2) predictors for having a continued high (chronic) level of LBP over longer periods of time. However, this has not previously been investigated in a larger working population.

The aim of this study was to examine these two aspects in a working population of 842 workers with repetitive measurements of LBP over one year.

**Methods:**

There were 842 workers from 15 companies in the DPhacto study participating in this study. Demographic, work- and health-related factors, and back endurance were measured at baseline, while 14 monthly repeated text message assessments of LBP intensity were prospectively collected. A factor analysis was used to cluster different time-patterns of LBP, and defining the group of participants with chronic LBP. A multi-adjusted logistic regression analysis was performed to investigate baseline predictors for chronic LBP.

**Results:**

The factor analysis revealed two dimensions of the time pattern of LBP, defined as the LBP intensity and LBP variation, respectively. A Visual Pain Mapping was formed based on the combination of the two pain dimensions, classifying the time-patterns of LBP into four categories: (1) low intensity and low variation, (2) low intensity and high variation, (3) high intensity and high variation, (4) high intensity and low variation (defined as chronic LBP). Significant baseline predictors for chronic LBP in the fully adjusted model were high baseline LBP (*p* < 0.01), low workability (*p* < 0.01), low BMI (*p* < 0.05), and being a blue-collar worker (vs. white-collar worker) (*p* < 0.05).

**Conclusion:**

This study presents a novel classification of the course of LBP based on repetitive measurements over a year, and revealed the predicting factors for chronic LBP based on repetitive measurements in a working population.

## Background

Musculoskeletal pain is a prevalent health issue in the working population. More than one million individuals have chronic musculoskeletal pain in Europe [1]. Low back pain (LBP) is among the leading causes of years with reduced life quality, physical activity limitation, work ability loss and absence from work worldwide [2–4]. Risk factors for developing [5–9] or not recovering [10] from LBP still remain unsettled, which hampers preventive strategies towards LBP in the general population.

An explanation for the inconsistent documentation on risk factors for LBP may be the way LBP is measured [11]. Previously, LBP was often based on surveys with recall periods up to one year which inherently disregarding fluctuations of LBP [12]. Such long recall periods are known to introduce severe risk of recall-bias [13]. To minimize recall bias, it is recommended that recall periods should not be longer than 1 month [14]. Repeated measurements of LBP at least once a month over longer periods are therefore required to elucidate the time course of LBP [15].

Until recently, methods for convenient sampling of repeated measurements of LBP over prolonged time have been lacking. However, mobile phones and text messages have been shown to convey a feasible, practical, inexpensive and well-accepted method to collect responses to regular brief questions of various health conditions, such as pain [16]. Text messages with frequent pain measurements may therefore provide a more valid pattern of LBP over a prolonged period (e.g. one year). However, such frequent measurements of pain provide large amounts of data and currently there are no standards established on how to process such data.

Frequent measurements of LBP have up till now been mainly conducted on patient populations [17], while cohort studies in non-patient populations using frequent LBP measurements are scarce. In non-patient populations, several time-patterns of LBP may be expected (e.g. sustained low levels, fluctuating levels and chronic high levels). To capture these different patterns, there is a need for easily computed and understandable categorizations based on the frequent measurements of LBP.

The aim of this study was therefore to investigate 1) the time pattern of LBP, and 2) predictors for chronic LBP with repetitive measurements of LBP over one year among 842 workers in the DPhacto study [18].

## Method

This study is conducted on frequently repeated prospective follow-up measurements of LBP and descriptive baseline characteristics from the Danish Physical Activity Cohort with Objective Measurements (DPhacto) [18]. The main purpose of the cohort was to investigate the association between physical activity at work and the pattern of musculoskeletal pain among blue-collar workers in a prospective design with frequent pain measurements. DPhacto was evaluated by The Research Ethics Committee of The Capital Region of Denmark (H-2-2012-011) and the Danish Data Protection Agency, and the protocol has been described in details elsewhere [18]. The companies in the DPhacto cohort were mainly recruited in collaboration with a labour union representative. And all participants provided informed written content prior to participate. The workers and management at the companies agreed upon that participation was voluntary, and would not influence the workers work status. Moreover, the management only attained information from the study on group level, not on the level of the individual worker.

### Study population

**Fig. 1:**
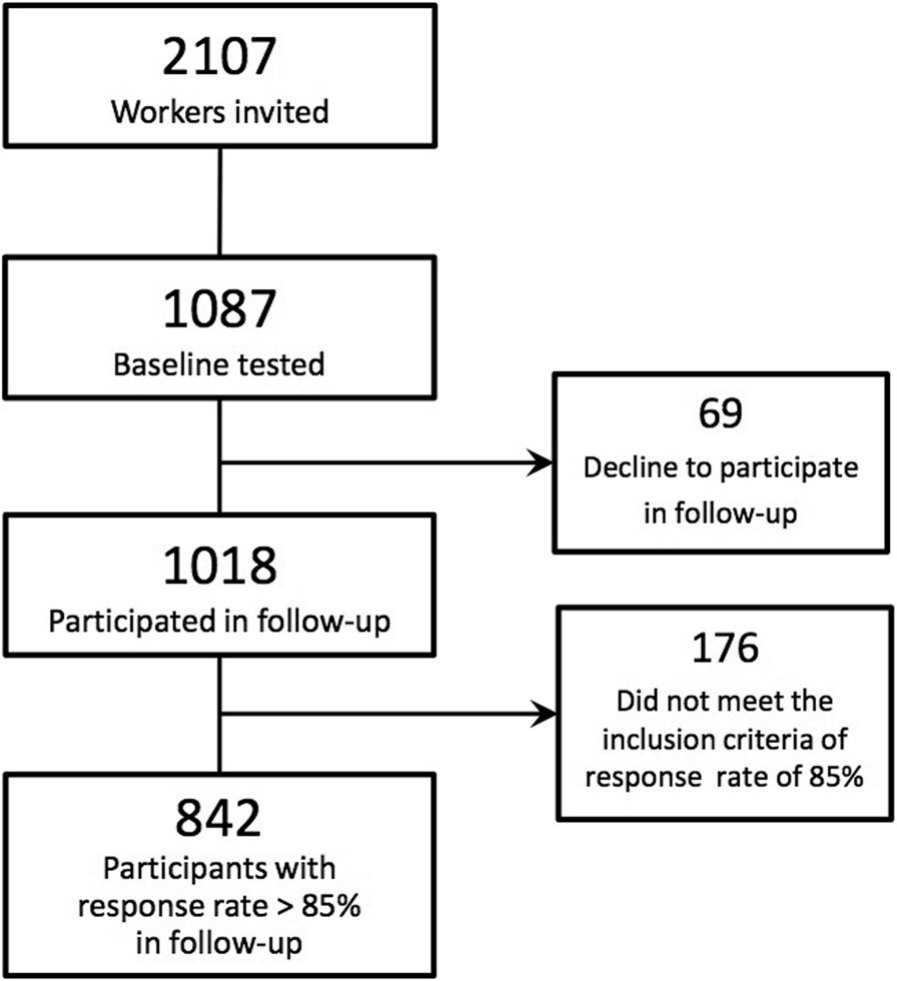
Flow diagram of the study population. Both blue- and white-collar workers from 15 different companies were invited to participate in the DPhacto study. Only workers who completed the follow-up on low back pain over one year with a response rate of at least 85 % were included in the analyses of the current study

Approximately 2000 workers from 15 different companies in the manufacturing, cleaning and transportation sector in Denmark were invited to participate in the study. The flow of the recruitment of the study population is illustrated in Fig. 1. Blue-collar workers with a variety of physically demanding work tasks were the primary target population. However, also administrative white-collar workers in 12 of the 15 companies were offered participation. Both blue- and white-collar workers were included in the analyses.

### Data collection

Data were collected from April 2012 to May 2014 with stepwise inclusion of companies. Workers were invited to participate in one-hour baseline measurements carried out by trained research personnel at the specific worksites during working hours. The baseline measurements included a computer based questionnaire and physical examination. Subsequently, workers were invited to participate in a one-year follow up on musculoskeletal pain occurrence and intensity.

### Baseline questionnaire

The questionnaire is described in detail in a previous paper [18]. It included questions regarding socio-demographic measures (e.g. gender, age, country of birth), education, work and employment along with physical and mental health. The present paper focuses on the questions and response options described in the following.

Employment status was categorically evaluated by the question “*Are you employed as blue-collar or white-collar worker?”*. Seniority was evaluated by the question “*For how long time have you had the kind of occupation as you have now? Respond in years and months”*. Answers were recalculated to total number of months. Work ability was evaluated by the question *“Please rate your present work ability?”* [19] rated on a scale from 0–10, with 0 being the worst and 10 being the best work ability. Physical strain at work was evaluated by the question “*How physically demanding do you normally consider your present work?”* [20], and rated on a scale from 1–10, with 1 being the least and 10 the most demanding work. Finally, LBP at baseline was evaluated by the question *“On a scale of 0–10, grade the worst pain you have experienced in your lower back within the past three months*? (Modified from [12]) and rated on a scale from 0–10, with 0 being no pain and 10 the worst possible pain”

### Physical examination

The physical examination included measurements of body height, body mass and a back endurance test [21]. BMI was calculated from body height and mass (kg/m^2^). In the back endurance test, participants were asked to lie prone on a plinth on the floor and lift and hold their upper body free from the floor for as long as possible. Workers reported extensive back pain 7 days prior to testing could choose not to participate in this particular test.

### Frequent prospective measurements on musculoskeletal pain intensity

Musculoskeletal pain intensity was collected by text messages every fourth week during the one-year follow-up period (i.e. 14 repeated assessments of LBP over one year). First, workers received a message introducing that the research questions were about to be sent to them, immediately followed by a text message with the question (“On a scale of 0–10, grade the worst pain you have experienced in your lower back within the past month? (0 = no pain, 10 = worst possible pain)” (modified from [12]). Participants received the first round of questions in the week of baseline measurement, followed by 13 additional rounds covering the one-year of follow-up. The questions were given on Sundays with a reminder on Mondays. If the reminder was not answered, personal follow-up by telephone calls was attempted at least three times during the following week. Distribution of questions and registration of answers were handled by trained research personnel using the Internet based software “SMS-Track®” (https://sms-track.com).

### Inclusion/exclusion criteria

For inclusion in the analyses for the present paper, workers were required to answer at least 12 (85 %) of the 14 follow-up questions of low back pain. Twelve out of 14 answers were considered as necessary to properly reveal the pattern and variation of LBP during the follow-up period. Workers were excluded during the one-year follow-up if they chose to terminate participation in the project or left the workplace.

### Data analyses

If answers were given in text (instead of numbers, as required), it was manually recoded into an adequate number. For example the answer “I have had no pain in the past month” was recoded as 0 and “My pain has been five on a scale from 0 to 10” was recoded as 5. If the text was insufficient for valid interpretation, the answer was recoded to missing. All cleaning of data were performed by trained research personnel and verified by a data manager.

### Statistical analyses

IBM SPSS Statistics 20 was used for processing and cleaning of all data as well as some basic descriptive statistics. SAS Enterprise Guide 5.1 was used for additional calculations and all statistical analyses.

Simple, descriptive statistical approaches (Table 1) were used for reducing the 12–14 repeated measures of LBP intensity for each worker to eight constructed pain variables: accumulation of LBP (summation of all pain intensity scores for each worker), mean LBP, median LBP, number of months with LBP assessments higher than 2 (LBP > 2) [22], number of months with LBP assessments higher than 4 (LBP > 4) [22], number of months with LBP assessments equal to 0 (LBP = 0), standard deviation and the variation span (numeric difference between the workers highest and lowest pain intensity rating). Table 1 presents the eight constructed pain variables and descriptive characteristics of the study population.

**Table 1:** Descriptive characteristic of the eight constructed variables of low back pain (*N* = 842)

Pain variables	Mean	(SD)	Range
Accumulated LBP^a^	32.5	(28.8)	0–137
Mean LBP	2.4	(2.1)	0–9.8
Median LBP	2.1	(2.3)	0–10
LBP > 2	5.5	(5.1)	0–14
LBP > 4	2.9	(4.1)	0–14
LBP = 0	5.2	(5.2)	0–14
Standard deviation	1.3	(0.8)	0–4.3
Variation span^b^	4.2	(2.7)	0–10

The eight constructed low back pain variables are calculated from the 12–14 repeated measures of low back pain intensity for each worker included from the DPhacto. LBP = low back pain. SD = Standard deviation. Mean and SD presents mean LBP intensity of the eight pain variables

^a^Accumulated LBP = summation of all LBP intensity scores for each worker

^b^ Variation span = numeric difference between the workers highest and lowest LBP intensity rating

A Pearson correlation analysis was used to investigate the correspondence between the pain variables.

To further reduce and simplify the data, a factor analysis was applied. The factor analysis reduces the eight pain variables to two (independent) factors. Only these two factors were retained for rotation in an orthogonal varimax rotation, ensuring that the factors were uncorrelated for easier interpretation.

The allocation of the pain variables by the correlation and the factor analysis were used to describe the respective factors. The factors were treated independently from each other, and were made normally distributed with mean = 0 and spread = 1. A factor was accepted as an independent factor if having an eigenvalue > 1.

**Fig. 2:**
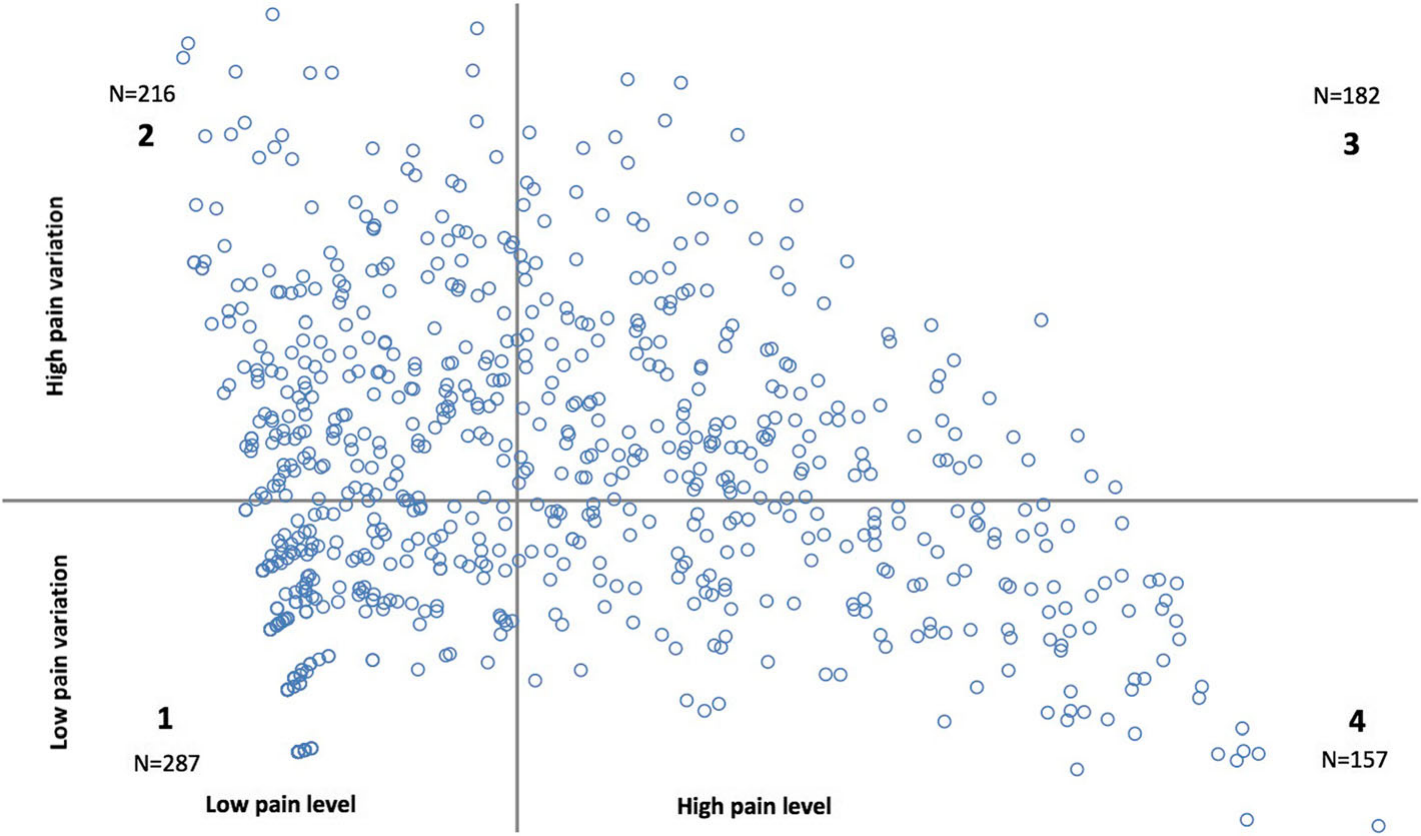
Visual Pain Mapping presenting the distribution of workers in the 4 pain categories ﻿in Dphacto. The Visual Pain Mapping is divided in following 4 categories: (1) ‘Low Pain Level and low Pain Variation’, (2) ‘Low Pain Level and high Pain Variation’, (3) ‘High Pain Level and high Pain Variation’ and (4) ‘High Pain Level and low Pain Variation’. N reports the number of workers in each category

Furthermore, the dimensions of pain defined by the factor analysis were combined in a constructed Visual Pain Mapping (Fig. 2). The Visual Pain Mapping is a summarized graphical description of the LBP experience of each participant during the one-year follow up period. The mapping is based on the factor values of LBP extracted from the population, and not depending on an apriority determined cut-point of LBP intensity.

Logistic regression was applied to test the association between baseline demographic variables and the LBP dimensions estimated from the repetitive LBP measurements over the following year. Three models were tested: model 1: Crude analysis, model 2: Adjusted for age and gender, model 3: Adjusted for age, gender, BMI, back endurance, baseline LBP intensity, sector, seniority, workability and physical strain in work. Due to multicollinearity, the model is not adjusted for position (i.e. being blue-collar or white-collar worker).

Odds ratios were considered as statistically significant of *p* < 0.05.

## Results

### Study population

From the invited 2107 workers, 1087 blue- and white-collar workers provided informed consent and participated in baseline measurements, and 1018 engaged in the one-year follow-up on musculoskeletal pain by text messages (Fig. 1). Sixty-nine workers declined to participate in the follow-up on musculoskeletal pain due to lack of interest, not having a mobile phone or being unable to handle text messages.

The majority of workers (62 %) completed the follow-up on LBP with a response rate of 100 %. Among the participants included in the follow-up, 3 % did not respond to any text messages, and 10 % responded to less than half of the text messages.

The study population included in the statistical analyses were 842 workers (82 %), who met the inclusion criteria of answering 12 or more of the 14 received questions (Fig. 1).

The 842 workers responding to 12–14 questions about LBP intensity provided a total of 11,511 assessments of LBP over the one-year period. As expected in a general working population, the assessments are highly orientated towards low LBP intensity with 37 % of the answers being 0, and 69 % of the answers being less than 4 on the LBP intensity scale from 0 to 10.

Characteristics of the population are presented in Table 2. The 842 workers have an equal gender distribution and an average age of 45 years. The majority of the population is blue-collar workers, and the manufacturing sector is strongly represented with 72 %. Seniority ranged from workers just being hired to workers with seniority of 45 years.

**Table 2:** Descriptive characteristics of the study population ﻿in Dphacto, *N* = 842

Descriptive characteristics	*n*	Mean (SD) or %
Age (Years), mean (SD)	842	45 (9.3)
Gender (% male)	842	51
Country of birth (% Denmark)	825	94
Sector (%)	842	
Manufacturing	602	72
Cleaning	164	19
Transport	76	9
Position (%blue-collar)	842	82
Seniority (Years), mean (SD)	816	13.3 (10.2)
Work ability (1–10)^a^, mean (SD)	837	8.4 (1.5)
Physical strain at work (0–10)^a^, mean (SD)	813	5.3 (2.4)
BMI (kg/m2), mean (SD)	821	27 (4.8)
Back endurance (Sec.)^b^, mean (SD)	544	106 (56.8)
LBP intensity baseline (0–10), mean (SD)	837	3.3 (3)

*SD* standard deviation, *BMI* Body Mass Index, *LBP* low back pain

^a^High workability and low physical strain at work is preferable

^b^Longer back endurance the better

### Grouping of the pain variables using factor and correlation analyses

The factor analysis was performed on the eight extracted LBP variables, and resulted in two factors with eigenvalues > 1, suggesting a two factor distribution. The eigenvalues for dividing into two factors were 1.5, with a degree of explanation of 92 %.

The factor analysis is supported by the correlation analysis between the same eight extracted LBP variables (Table 3). The correlation analysis revealed fairly high agreement between accumulated LBP, mean LBP, median LBP and LBP > 2, LBP > 4, and LBP = 0, with correlations ranging from 0.99 – 0.57. These LBP variables were all poorly correlated with the two variables: standard deviation and variation span, with 0.46 as the highest correlation. However, the standard deviation and the variation span were highly correlated with each other, with a correlation of 0.96.

**Table 3:** Correlation matrix between the eight constructed variables of low back pain (LBP) ﻿in Dphacto, *N* = 842 (*p* < 0.001)

	Accumulated LBP	Mean LBP	Median LBP	LBP >2	LBP >4	LBP =0	Standard Deviation	Variation span
Accumulated LBP	1	-	-	-	-	-	-	-
Mean LBP	0.99	1	-	-	-	-	-	-
Median LBP	0.97	0.98	1	-	-	-	-	-
LBP > 2	0.94	0.93	0.91	1	-	-	-	-
LBP > 4	0.92	0.92	0.89	0.81	1	-	-	-
LBP = 0	−0.81	−0.81	−0.79	−0.80	−0.57	1	-	-
Standard deviation	0.42	0.42	0.31	0.38	0.32	−0.42	1	-
Variation span	0.43	0.43	0.33	0.40	0.32	−0.46	0.96	1

Analysed with Pearson correlation. LBP = low back pain. The eight LBP variables are constructed for all blue- and white-collar workers included from the DPhacto study. *Accumulated LBP*: summation of all LBP intensity scores for each worker, *mean LBP*: the mean of all LBP intensity scores, *median LBP*: the median of all LBP intensity scores, *LBP > 2*: number of months with LBP assessments higher than 2, *LBP > 4*: number of months with LBP assessments higher than 4, *LBP = 0*: number of months with LBP assessments equal to 0, *standard deviation* of all LBP intensity scores, and the *variation span*: numeric difference between the workers highest and lowest pain intensity scores. Coefficients are shown between all eight LBP variables with *p* < 0.001

Therefore, the factor analysis and the correlation analysis suggest a clear grouping of the eight LBP variables into two meaningful dimensions. Each dimension was given an explanatory title: “*Pain Level*” (i.e. comprising the LBP variables: accumulated LBP, mean LBP, median LBP, LBP < 2, LBP < 4 and LBP = 0) and “*Pain Variation*” (i.e. comprising the LBP variables: standard deviation and variation span).

### Visual pain mapping

The two dimensions; *Pain Level* and *Pain Variation* were combined in a Visual Pain Mapping (Fig. 2). The first factor representing *Pain Level* defines the x-axis and the second factor representing *Pain Variation* defines the y-axis. The axes crosses at the factor value 0; their mean values, thereby dividing the Visual Pain Mapping into the following four categories:

1. Low *Pain Level* and low *Pain Variation*: the workers generally having no or a low LBP intensity.
2. Low *Pain Level* and high *Pain Variation*: the workers generally having no or low LBP intensity, but occasionally experiencing episodes with higher levels of LBP.
3. High *Pain Level* and high *Pain Variation*: the workers generally having high intensity of LBP, but occasionally experience episodes with low or no LBP.
4. High *Pain Level* and low *Pain Variation*: the workers who generally have a sustained high LBP intensity, which is defined in this study as having chronic LBP.

The visual pain mapping presents the distribution of workers within the four categories of LBP based on the coordinates from their two factor values. Since the two factors are normally distributed, the population should mainly divide equally between the four categories. Nevertheless, the allocation of the workers is slightly skewed. Category 1 contains the highest number of workers (*N* = 287), but also presents the smallest spread (Fig. 2). This is due to the skewed distribution of LBP ratings in this population with many workers generally having no or a low LBP level during the follow-up period. In category 2 and 4 the spread of the distribution of workers are rather large because of the capturing of the extreme cases; workers who experience either a large variation imposed on a general low pain level (top left of category 2) and workers with constant high pain level (right bottom of category 4). Category 3 represents the workers generally having high intensity of LBP, but occasionally experience episodes with low or no LBP. In this category there are no extreme cases in the outer corner of the map.

Descriptive characteristics for each of the four pain categories are presented in Table 4. As expected, the baseline characteristics tend to differ between the four categories. Males are overrepresented in the lower pain categories and females are overrepresented in the higher pain categories, but there is a large sector-related gender distribution with most females in cleaning and most males in transportation and workers from the cleaning sector seem to be overrepresented in the higher pain categories. Blue-collar workers show similar tendencies as cleaners, while the distribution of white-collar workers is highly skewed towards the lower pain categories. Workers with high work ability, low physical strain at work, high back endurance and low LBP intensity at baseline tend to be overrepresented in category 1, and correspondingly the workers with the opposite characteristics tend to be overrepresented in category 4. There is a small tendency of more workers with BMI < 25 to be located in category 1, compared to workers with higher BMI. No clear tendencies are seen for seniority.

**Table 4:** Distribution of baseline demographics in the four generated categories of low back pain ﻿in Dphacto, *N* = 842

		Low intensity Low variation	Low intensity High variation	High intensity High variation	High intensity Low variation
		*n* = 287 (34 %)	*n* = 216 (26 %)	*n* = 182 (22 %)	*n* = 157 (19 %)
	*n*	%	%	%	%
Age (years), mean (SD)	842	45 (9)	44 (10)	46 (9)	47 (9)
Gender	842				
Male	430	36	27	20	18
Female	412	32	24	24	20
Sector	842				
Manufacturing	602	35	26	22	18
Cleaning	164	30	23	24	23
Transport	76	37	30	14	18
Position	842				
Blue-collar	693	32	25	22	21
White-collar	149	44	30	19	7
Seniority	816				
0–5 years	178	34	29	19	17
5–10 years	192	33	30	20	16
10–20 years	220	35	22	26	17
> 20 years	226	34	23	20	23
Workability^a^	842				
9–10	468	43	26	19	12
8	211	29	25	24	22
0–7	163	15	26	26	33
Physical strain at work^a^	813				
1–3	224	42	29	18	11
4–7	415	35	24	21	20
8–10	174	22	25	28	25
BMI (kg/m2)	842				
< 25	306	36	26	18	20
25–30	320	35	25	22	18
> 30	216	30	26	26	18
Back endurance^b^	842				
= > 90 sec	326	45	28	16	11
46–89 sec	89	27	38	17	18
11–45 sec	124	33	28	19	20
< 10 sec	303	25	18	30	27
LBP intensity baseline	837				
0	257	61	27	7	5
1–4	295	36	28	20	15
5–7	175	11	25	30	34
8–10	110	2	18	47	33

Demographical distribution of the four categories of LBP: (1) ‘Low Pain Level and low Pain Variation’, (2) ‘Low Pain Level and high Pain Variation’, (3) ‘High Pain Level and high Pain Variation’ and (4) ‘High Pain Level and low Pain Variation’

*SD* standard deviation, *BMI* body mass index, *LBP* low back pain

^a^High workability and low physical strain at work is preferable

^b^The longer back endurance the better

### Baseline risk factors for chronic LBP

The logistic regression analysis, presented in Table 5, shows the baseline risk factors for having chronic LBP (LBP category 4). In the fully adjusted model, workers with high LBP intensity at baseline (i.e. the two groups with 5–7 and 8–10) had significantly elevated odds ratios (OR: 7.85 and 6.52) for chronic LBP compared to workers with no LBP at baseline.

**Table 5:** Baseline predictors for chronic low back pain (LBP) ﻿in Dphacto, *N* = 842

		Model 1		Model 2		Model 3	
Demographic factors	*n*	OR (95 % CI)	*P*-value	OR (95 % CI)	*P*-value	OR (95 % CI)	*P*-value
Age (years)	842	1.03 (1.00–1.05)	**0.01**	1.03 (1.01–1.05)	**0.01**	1.01 (0.99–1.04)	0.29
Gender	842						
Male	430	REF		REF		REF	
Female	412	1.14 (0.81–1.61)	0.46	1.08 (0.76–1.54)	0.65	1.17 (0.75–1.83)	0.49
Sector	842						
Manufacturing	602	REF		REF		REF	
Cleaning	164	1.36 (0.89–2.08)	0.25	1.25 (0.79–1.98)	0.46	1.05 (0.63–1.77)	0.83
Transport	76	1.06 (0.57–1.96)	0.75	1.04 (0.55–1.98)	0.83	0.97 (0.47–2.01)	0.89
Position	842						
Blue-collar	693	REF		REF		REF	
White-collar	149	0.30 (0.16–0.57)	**<.01**	0.30 (0.16–0.56)	**<.01**	0.46 (0.21–0.99)	**<0.05**
Seniority	816						
0–5 years	178	REF		REF		REF	
5–10 years	192	0.91 (0.53–1.58)	0.41	0.88 (0.51–1.52)	0.60	0.92 (0.50–1.68)	0.54
10–20 years	220	0.96 (0.57–1.62)	0.57	0.85 (0.50–1.45)	0.45	0.90 (0.49–1.64)	0.45
> 20 years	226	1.38 (0.84–2.27)	0.06	1.13 (0.66–1.93)	0.30	1.34 (0.72–2.48)	0.14
Workability	842						
9–10	469	REF		REF		REF	
8	211	2.11 (1.37–3.23)	0.62	2.11 (1.38–3.25)	0.59	1.70 (1.06–2.71)	0.68
0–7	163	3.65 (2.37–5.60)	**<.01**	3.61 (2.34–5.56)	**<.01**	2.41 (1.49–3.89)	**<0.01**
Physical strain at work	813						
1–3	224	REF		REF		REF	
4–7	415	2.05 (1.26–3.34)	0.25	2.06 (1.27–3.37)	0.22	1.17 (0.64–2.12)	0.93
8–10	174	2.74 (1.59–4.72)	**<.01**	2.69 (1.55–4.67)	**<.01**	1.41 (0.72–2.77)	0.29
BMI (kg/m2)	842						
< 25	306	REF		REF		REF	
25–30	320	0.91 (0.61–1.36)	0.80	0.90 (0.60–1.34)	0.80	0.73 (0.46–1.15)	0.96
> 30	216	0.90 (0.58–1.41)	0.79	0.88 (0.56–1.38)	0.72	0.54 (0.32–0.90)	**<0.05**
Back endurance	842						
= > 90 sec	326	REF		REF		REF	
46–89 sec	89	1.82 (0.96–3.47)	0.95	1.80 (0.94–3.43)	0.99	1.56 (0.78–3.12)	0.91
11–45 sec	124	2.10 (1.20–3.68)	0.50	2.05 (1.17–3.61)	0.48	1.76 (0.93–3.35)	0.48
< 10 sec	303	3.03 (1.97–4.68)	**<.01**	2.81 (1.80–4.39)	**<.01**	1.92 (1.16–3.19)	0.14
LBP intensity baseline	837						
0	257	REF		REF		REF	
1–4	295	3.12 (1.76–5.84)	0.14	3.26 (1.74–6.10)	0.19	3.28 (1.71–6.27)	0.57
5–7	175	9.06 (4.86–16.88)	**<.01**	9.24 (4.95–17.27)	**<.01**	7.85 (4.11–14.99)	**<.01**
8–10	110	8.44 (4.32–16.50)	**<.01**	8.40 (4.29–16.46)	**<.01**	6.52 (3.19–13.33)	**<.01**

The four categories of low back pain (LBP) defined as: (1) ‘Low Pain Level and low Pain Variation’, (2) ‘Low Pain Level and high Pain Variation’, (3) ‘High Pain Level and high Pain Variation’ and (4) ‘High Pain Level and low Pain Variation’ (chronic LBP). Analyses are made with logistic regression based on all blue- and white-collar workers included. OR = Odds ratio, CI = Confidence limits, REF = Reference group, BMI = Body Mass Index, LBP = Low back pain. Model 1: Crude analysis, Model 2: Adjusted for age and gender, Model 3: Adjusted for age, gender, BMI, back endurance, baseline LBP intensity, sector, seniority, workability and physical strain in work. Significance level is set at p>0.05

Moreover, workers with low work ability (0–7) had a significantly increased odds ratio (OR: 2.41) for chronic LBP compared to workers with high work ability (9–10). White-collar workers were found to have a significantly lower odds ratio (OR: 0.46) for chronic LBP compared to blue-collar workers. No significant odds ratios were found for physical strain at work, back endurance, age, gender and seniority in model 3. Surprisingly, workers with high BMI (>30) were observed to have significantly lower odds ratio (OR: 0.54) for chronic LBP compared to workers with low BMI (<25).

## Discussion

The aim of this prospective study was to investigate the time pattern of LBP and baseline predictors for chronic LBP with repetitive measurements of LBP over one year in a working population. A factor analysis, based on eight LBP variables constructed from the repeatedly measured LBP, provided two main dimensions: *pain level* and *pain variation*. The two dimensions were transformed into a Visual Pain Mapping disclosing four categories describing the experienced pain over the past year with follow-up measures. This methodological approach identified the group of workers with high pain and low variation, termed as chronic LBP. The main significant baseline characteristics increasing the risk of being classified with chronic LBP were low work ability, high baseline LBP, the position as a blue-collar worker and surprisingly, also low BMI.

The factor analysis indicates that the two dimensions of pain level and pain variation as well as the four distinct combinations (i.e. the combination of high and low of pain level and pain variation) can be used to categorize pain patterns based on one-year registrations. Previous studies have typically used clusters [23], trajectories [24] or pattern recognition [3, 25] to categorize and describe pain categories. These methods all differ from the factor analysis used in the present study as they typically define theoretically based definitions of the different categories, to classify individuals. The factor analysis is an easily conducted statistical approach based on data from the study population, which in this case provided theoretically sound classifications of the population based on the repetitive measurements of LBP.

The present study is conducted among a working population with no pre-specified assumptions of LBP intensity level. Previous studies on the course of LBP have typically been conducted on patient populations, who are diagnosed, based on pain intensity at baseline and subsequently have received treatment during the follow-up period [26]. Thus patients in these studies are, compared to the present study, pre-classified by having initial pain high enough to seek professional care. Moreover, the patients receive treatment, which together with the regression towards the mean provide a strong hypothesis that their pain intensity level will decrease during the follow-up, thereby providing a template for a pattern based classification. This is not comparable to the present study, which is novel in describing LBP over a longer period in a working population with a large variety of LBP. As the workers do not have a common starting point or follow a specific pattern of development over time, a different approach was needed to properly classify the working population and identify the workers that may be at risk of aggravating towards chronic LBP. The Visual Pain Mapping, formed by the two LBP dimensions; pain level and pain variation, potentially seems to be a useful tool for this categorization and description of the workers in a selected population based on their LBP intensity ratings.

### Baseline predictors for chronic LBP

Workers, with the baseline descriptive characteristics of high LBP intensity, low work ability or were blue-collar workers, had a significantly increased risk (model 3) of presenting with a chronic pain pattern in the one-year prospective follow-up. This corresponds well with known risk factors in the literature [27–29]. Moreover, in contrast to what was expected, we found a tendency of a decrease in risk with higher BMI. This association was significant for high BMI (>30) in model 3. No differences between the BMI groups were found regarding gender, age, seniority, work ability, physical strain in work, baseline LBP, gender, sector or position. Only back endurance tended to a difference with longer endurance in the low BMI group (133 s.) then in the high BMI group (69 s.). Since a high endurance could be supposed to protect from pain this difference does not offer an explanation for the higher risk of LBP in the low BMI group. Surprisingly, we found no clear indication of gender or seniority being a risk factor.

### Strengths and limitations

A main strength of this study with repetitive pain measures is the novelty in the choice of population of workers distinguished from the literature mainly conducted among patients. Furthermore, the high response rates in this study ensure a reliable picture of the natural course of low back pain, as an essential premise for a correct classification.

A limitation to this type of analyses is the highly skewed data towards no or low pain creating a floor effect of the data. This prevents a true normal distribution of the two factors and an even distribution of the workers in the 4 categories. The cut-points between high and low pain level and variation are, as previously described, statistically founded based on the actual population and not relying on theoretical pain intensity considered clinically important based on other populations as often used in previous studies.

A limitation of the data on LBP in this study is that only intensity of LBP is recorded with no registration of duration [30]. It is therefore not possible to determine whether the reported LBP consists of multiple single pain events or one (or more) coherent periods. Furthermore, the variation within the month is also not investigated, which potentially could influence the assessment of LBP [31]. We therefore recommend future studies to also collect data on duration of pain and with an even higher frequency than the monthly recordings of this study.

### Practical implications

This study is explorative in the search to investigate characteristics of the time-pattern of LBP from repetitive measurements of LBP over one year in a working population. The presented novel method is easy to apply on repetitive data and is especially suited for use in both research and by practitioners working with prevention of LBP in the field. Future studies using frequent measurements of LBP are however required for documenting the time-pattern of LBP and predictors for chronic LBP in working populations. The Visual Pain Mapping is easy to apply on repetitive data and is especially suited for use by clinicians working with prevention of LBP in the field.

Prospectively, a next step could be to analyse if the chronic pain category has a predictive validity with respect to sickness absence and premature dropout from the labour market.

## Conclusion

This study provides novel information based on repetitive measurements on the one-year natural course of LBP in a working population presented as a Visual Pain Mapping. Moreover, we found that the predicting factors for chronic LBP were high baseline LBP, low work ability, low BMI, and being a blue-collar worker. More studies using frequent measurements of LBP are required for further documenting the time-pattern of LBP and predictors for chronic LBP in working populations.

## Abbreviations

DPhacto: Danish physical activity cohort with objective measurements
LBP: Low back pain
